# The complete chloroplast genome sequence of *Liparis bootanensis* (Orchidaceae)

**DOI:** 10.1080/23802359.2020.1763866

**Published:** 2020-05-14

**Authors:** Jiang-Feng Liu

**Affiliations:** Management Office of Yushan Scenic Park, Fuzhou, China

**Keywords:** Chloroplast genome, Illumina sequencing, phylogeny, *Liparis bootanensis*

## Abstract

*Liparis bootanensis* is an epiphytic orchid distributed in tropical and subtropical regions of Asia, and has been listed as an endangered species in the Wildlife Conservation List. In this study, the complete chloroplast genome of *L. bootanensis* was assembled using Illumina sequencing data. The complete chloroplast (cp) genome is 158,325 bp in length, including a pair of invert repeats (IRA and IRB) regions of 26,700 bp, large single-copy (LSC) region of 86,584 bp, and small single-copy (SSC) region of 18,341 bp. The chloroplast genome contains 133 genes, including 83 protein-coding genes, 38 tRNA genes, and 8 rRNA genes. The phylogenetic analysis indicated that *Oberonia japonica* was closely related to *L. bootanensis* based on 17chloroplast genomes matrix of Orchidaceae.

The genus *Liparis* (Orchidaceae) comprises about 400 species and is widely distributed in the tropical and subtropical regions of Asia, Oceania and Americas, with only a few extending to the temperate zone (Chen et al. 2009; Lee et al. 2010; Lin and Yan 2013). *Liparis* species can be terrestrial, epiphytic or lithophytic (Chen et al. 2009). Morphologically, *Liparis* is characterized by resupinate flowers and curved column. *Liparis bootanensis* is an epiphytic orchid and widely distributed in tropical and subtropical regions of Asia. It usually grows on the tree trunk in forest and rocks or cliffs along valleys (Chen et al. 2009). It has been used in folk medicine in China (Liang et al. 2019). Due to the excessive collection and habitat destruction, the population size of *L. bootanensis* has declined during the last several decades. In this study, the complete chloroplast genome of *L. bootanensis* was assembled using Illumina sequencing data, which would be helpful for the formulation of conservation strategies and further research of *L. bootanensis.*

The total genomic DNA was extracted from fresh leaves using the modified CTAB method (Doyle and Doyle 1987) and sequenced based on the Illumina pair-end technology. The leaf sample of *L. bootanensis* was collected from Qinglong waterfall scenic area, Yongtai, Fujian province, China (25°46′N, 118°57′E). The voucher specimen is kept at the Herbarium of Fujian Agriculture and Forestry University (specimen code FAFU03805). Approximately 10 Gb of sequences data were extracted from the total sequencing output and input into Organelle PBA (Soorni et al. 2017) to assemble the chloroplast genome. Annotation of the chloroplast genome was performed using the Dual Organellar GenoMe Annotator (DOGMA) online tool (Wyman et al. 2004) and Geneious ver. 2019.1.1 (Li et al., 2019), then manually verified and corrected by comparison with *L. loeselii* (GenBank accession MF374688). Finally, we obtained a complete chloroplast genome of *L. bootanensis* and submitted to GenBank with an accession number (MN627759).

The total chloroplast genome sequence of *L. bootanensis*is 158,325 bp in length with GC content of 36.9%. It contains a pair of inverted repeats (IR) regions of 26,700 bp, a large single-copy (LSC) region of 86,584 bp, and a small single-copy (SSC) region of 18,341 bp.The chloroplast genome has a total of 133 genes, including 83 protein-coding genes, 38 tRNA genes, and 8 rRNA genes.

To explore the phylogenetic position of *L. bootanensis*, 16 complete chloroplast genomes of Orchidaceae (*Apostasia odorata*, *A. shenzhenica*, *A. wallichii*, *Bletillastriata*, *B. ochracea*, *Pleione bulbocodioides*, *Corallorhizabulbosa*, *C. macrantha*, *Eulophia zollingeri*, *Dendrobium aphyllum*, *D. aduncum*, *L. loeselii, Oberonia japonica*, *Vanilla planifolia*) were used to perform phylogenetic analysis. All the sequences were downloaded from NCBI GenBank. The sequences were aligned by MAFFT v7.307 (Katoh and Standley 2013), and the phylogenetic tree was constructed by RAxML (Stamatakis 2014). The results showed that *O. japonica* was most closely related to *L. bootanensis* (Figure 1).

**Figure 1.**
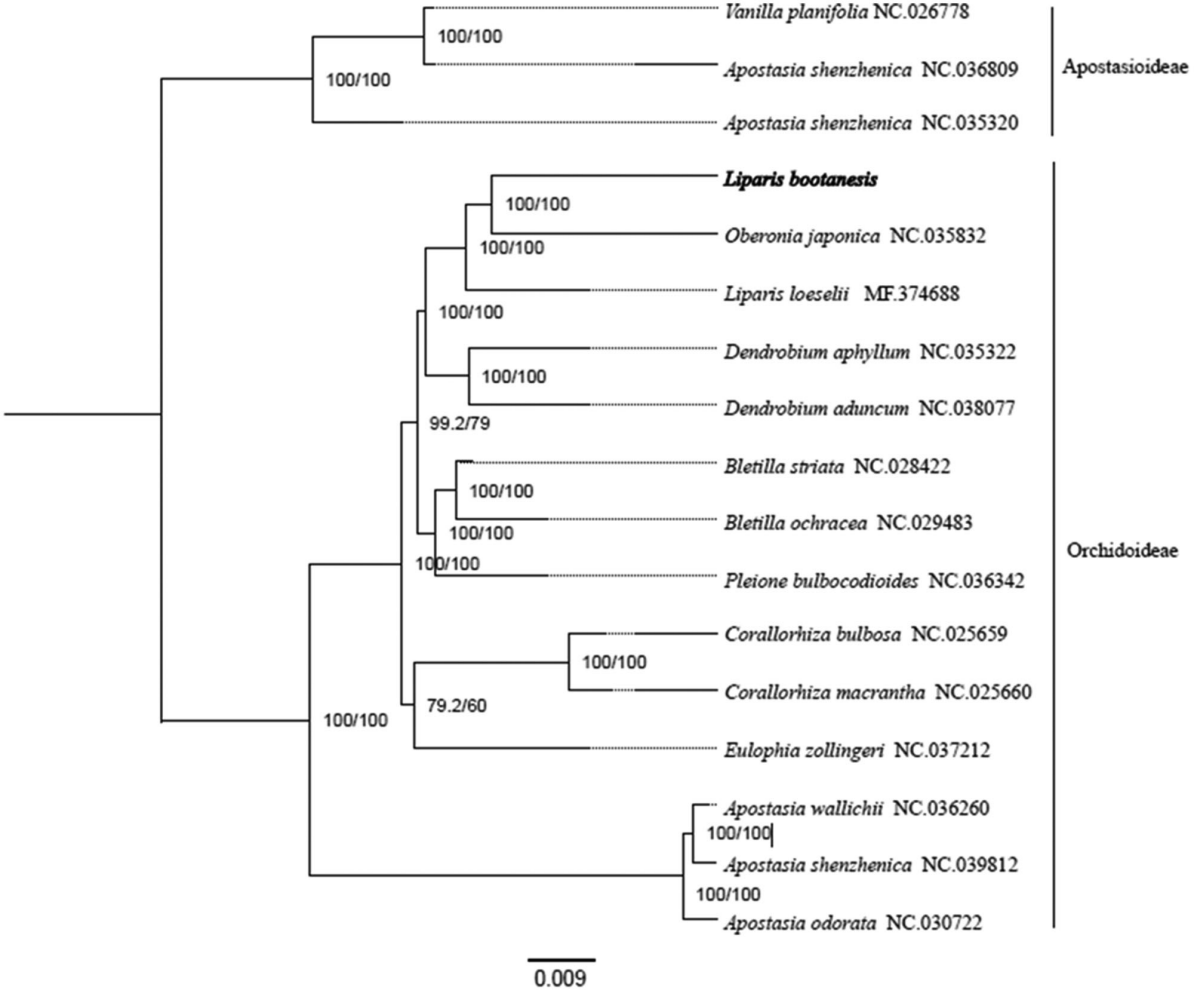
Phylogenetic tree based on 17 complete chloroplast genome sequences of Orchidaceae.

## Data Availability

Data openly available in a public repository that does not issue DOIs. The data that support the findings of this study are openly available in [National Center for Biotechnology Information] at [https://www.ncbi.nlm.nih.gov/], reference number [MN627759].
